# B cells and iBALT in TB immunity & pathogenesis

**DOI:** 10.3389/fimmu.2026.1743572

**Published:** 2026-02-04

**Authors:** Taru S. Dutt, Robert Krause, David Hertz, Marcela Henao-Tamayo, Alasdair Leslie, Bianca Schneider

**Affiliations:** 1 Division of Laboratory Medicine, Department of Pathology, Heersink School of Medicine, University of Alabama at Birmingham, Birmingham, AL, United States; 2 Africa Health Research Institute, School of Laboratory Medicine and Medical Sciences, University of KwaZulu-Natal, Durban, South Africa; 3 Host Determinants in Lung Infections, Priority Research Area Infections, Research Center Borstel, Leibniz Lung Center, Borstel, Germany; 4 Department of Microbiology, Immunology, and Pathology, College of Veterinary Medicine and Biomedical Sciences, Colorado State University, Fort Collins, CO, United States; 5 Department of Infection and Immunity, University College London, London, United Kingdom

**Keywords:** antibodies, B cells, iBALT, sex differences, TLS, tuberculosis

## Abstract

B cells play a crucial role in immunity against various infectious diseases. However, their role in tuberculosis (TB) has been largely understudied. Emerging evidence suggests that B cells actively shape immune responses in TB. Beyond their classical functions, B cells contribute to the formation of inducible bronchus-associated lymphoid tissue (iBALT), a tertiary lymphoid structure (TLS) that enhances localized immune responses in the lungs. As iBALT is a site for B-T cell interactions and the generation of high-affinity antibodies, recent studies suggest that sex differences in iBALT formation influence TB immunity. This review synthesizes evidence from both TB and non-TB models to highlight the expanding role of B cells and iBALT, underscoring their potential implications for vaccine development and immunotherapy.

## 1 Introduction

Tuberculosis (TB), caused by *Mycobacterium tuberculosis* (*Mtb*), remains a leading cause of infectious disease-related mortality worldwide (1). Despite extensive research efforts, developing an effective vaccine beyond Bacille Calmette-Guérin (BCG) continues to face substantial hurdles (2, 3). This challenge is partly due to the limited understanding of the mechanisms underlying protective immunity and the distinction between beneficial and pathogenic immune responses (4, 5). Given the intracellular nature of *Mtb*, TB research has traditionally centered on T cell-mediated immune responses (6–10). While T cells are undoubtedly essential, emerging evidence highlights the previously underappreciated role of B cells in TB immunity (11–16). Beyond their classical function of antibody production, B cells contribute significantly to antigen presentation (17), cytokine secretion (18, 19), and the formation of tertiary lymphoid structures (TLS), such as inducible bronchus-associated lymphoid tissue (iBALT) (16, 20–23). Notably, recent studies also suggest that the formation and function of iBALT can exhibit sex-specific differences, potentially influencing the progression and outcome of TB disease (24–27). Intriguingly, males are more susceptible than females according to global statistics. Sex hormones along with genetic factors, drive differences in immune responses between males and females, which may shape iBALT organization and its protective efficacy against *Mtb* infection (27). These findings suggest that B cells are not mere bystanders but central players in TB pathogenesis and protection, necessitating a reassessment of their role in shaping immune responses to *Mtb* infection, particularly in both sexes.

Historically, evaluating immune responses in TB, particularly the diversity and functional heterogeneity of B cells, their interactions with other immune cells, and their specific roles within the lungs—the primary site of infection—has been challenging. However, recent advancements in immunological techniques, including spatial transcriptomics and single-cell RNA sequencing, now allow for a more detailed characterization of the tissue microenvironment and the identification of distinct subsets of lung-resident B cells (14, 28, 29). This represents a new opportunity to unravel the role of B cells in localized immune response to *Mtb* infection; knowledge that will improve our understanding of the immune correlates of protection in TB and may inform the development of innovative therapeutic strategies. In this review we summarize these emerging insights, address current knowledge gaps and encourage deeper exploration into the diverse functions of B cells in TB immunity.

## 2 B cells and TB: what we know so far

Traditionally, B cells have been regarded as secondary players in the immune response to *Mtb*. However, over the past decade, numerous studies have challenged this view, revealing a potentially pivotal role for B cells in TB immunity (23, 30–33). During *Mtb* infection, B cells are reduced in peripheral blood and present within the granulomas in the lung (14, 32, 34–37), the hallmark structures of TB pathology. Moreover, B cells are well recognized for their canonical roles in immunity, such as antibody production (38); however, studies over the past decade have uncovered non-canonical functions of B cells that further expand their immunological significance (39). Through antigen presentation, cytokine production, and antibody secretion, B cells shape the local immune microenvironment and influence the dynamics of host-pathogen interactions (40, 41).

A particularly intriguing aspect of B cell activity in TB is their presence in TLS/iBALT. These structures emerge in response to chronic inflammation or infection and provide a localized lung environment where adaptive immunity is enhanced (42, 43). Here B cells also function as antigen-presenting cells (APCs), interacting with T cells to amplify immune responses (40, 44, 45). This underscores their importance in bridging humoral and cellular immunity to effectively combat *Mtb*.

A comprehensive understanding of both canonical and non-canonical roles is crucial to fully unravel the complexity of B cell-mediated immunity in TB. Here, we consolidate current knowledge on the diverse functions of B cells in TB and other infectious and non-infectious diseases, highlighting both established and emerging roles, with a focus on iBALT.

### 2.1 Canonical role of B cells

Antibodies can directly neutralize *Mtb* by binding to its surface antigens, thereby preventing bacterial adhesion to host cells and intracellular entry (30). Through opsonization, antibody-coated *Mtb* is internalized by macrophages via Fc receptor-mediated uptake, known as antibody-dependent cellular phagocytosis (ADCP), a process that promotes phagosome maturation and enhances bacterial clearance (46, 47). Studies have identified anti-lipoarabinomannan (LAM) antibodies as particularly effective in inhibiting *Mtb* growth within macrophages by enhancing opsonization and promoting phagosome maturation (48, 49). Moreover, these antibodies have been shown to promote bacterial clearance through complement activation (50, 51). Fc regions also induce antibody-dependent cellular cytotoxicity (ADCC) reactions through neutrophil and natural killer (NK) cell degranulation which eliminates infected cells and controls bacterial load (47). Additionally, antibodies against *Mtb*-secreted proteins, including Ag85, ESAT-6, and CFP-10, are thought to disrupt bacterial virulence mechanisms and enhance host defense. Intriguingly, individuals highly exposed to *Mtb* who did not develop classical latent TB infection (as defined by positive IFN-γ release assay or tuberculin skin test) exhibited *Mtb*-specific humoral immunity with enhanced avidity, a shift toward the IgG1 subclass, and distinct IgG Fc-glycosylation profiles, instead of the typical IFN-γ response (52). Importantly, differential Fc glycosylation was found to modulate Fc-mediated antibody activity, indicating its potential to directly impact *Mtb* control (30). Furthermore, the corresponding Fc receptors (FcRs) appear to shape the overall immune response to *Mtb*. Mice lacking activating FcRs develop more severe immunopathology and higher bacterial burdens, whereas mice deficient in the inhibitory FcR show reduced pathology (53). Collectively, these lines of evidence suggest that Fc-mediated antibody effector functions together with the expression of Fc receptors significantly contribute to the modulation of TB disease.

Despite these advances, the precise contribution of antibodies to protection against TB remains contentious. Passive transfer experiments have shown that antibodies can confer measurable protection in some settings (31, 54–56), yet other studies report minimal or no benefit, highlighting the complexity of humoral responses to *Mtb (*33, 57). The protective capacity of antibodies likely depends on multiple factors, including isotype, antigen specificity, and Fc receptor engagement. Recent developments in systems serology and high-throughput sequencing now enable detailed profiling of the TB-specific antibody repertoire, offering new insight into how distinct antibody subclasses and functions may contribute to protection.

### 2.2 Non-canonical role of B cells

While B cells are best known for their role in antibody-mediated immunity, they also perform several non-canonical, regulatory, or tissue-specific functions, which are increasingly recognized. Among these roles, cytokine production has emerged as a critical mechanism by which B cells shape both innate and adaptive responses. A specialized subset known as regulatory B cells (Bregs) is particularly enriched for IL-10 secretion, enabling these cells to suppress excessive inflammation and maintain immune homeostasis (58, 59). Bregs have been detected in the blood of individuals with active TB (58), and they are also present in the lungs of *Mtb*-infected mice, particularly during the chronic phase of infection (59). Recent findings indicate that the absence of B cell-derived IL-10 enhances resistance to TB, suggesting that B cell–specific IL-10 interferes with optimal infection control (24). B cells can also produce other immunoregulatory cytokines that may impact TB outcomes including IL-35, and IL-35/IL-20 co-producing B cells have been identified in TB (60). Complementing these observations, *in vivo* studies have shown that B cell derived IL-35 can suppress T cell responses, potentially undermining anti-*Mtb* immunity (61). Likewise, type I IFN producing B cells may contribute to impaired *Mtb* control by promoting macrophage polarization towards an anti-inflammatory phenotype (62). Conversely, Tbet expressing B cell can secrete IFN-γ, which is central to TB immunity, and marginal zone B cells secrete numerous cytokines in response to *Mtb* stimulation *ex vivo*, including TNF-a, IL-2 and GM-CSF (63). Although these cytokines play an important role in TB the relevance of their B cell source *in vivo* is not yet clear. Finally, Linge et al. also demonstrate that B cell-derived IL-6 production following *Mtb* infection exerts pleiotropic effects on both B and T cell function (64). Collectively, these findings indicate that cytokine-producing B cell subsets can exert both protective and potentially detrimental effects during *Mtb* infection, shaping the balance between host defense and pathogen persistence.

One of the most intriguing aspect of B cells in TB is their participation in the formation of iBALT, a type of TLS that develops in the lung (40). Unlike secondary lymphoid organs (SLOs) such as lymph nodes, spleen, tonsils, and Peyer’s patches, which are anatomically predetermined, TLS develop dynamically in response to localized immune challenges (65–67). These structures form directly at the site of *Mtb* infection, creating a highly specialized immune microenvironment that promotes antigen presentation, cytokine signaling, and the maintenance of immune memory (40).

## 3 Putative mechanisms underlying iBALT formation: role of inflammation, infection, and cytokines

Although the precise mechanisms underlying iBALT formation in TB remain undefined, data from other respiratory infections indicate that inflammation, antigenic stimulation, lymphotoxin (LT) signaling, cytokines, and chemokines are key drivers of its development (68–71). Pro-inflammatory cytokines such as interleukin-17 (IL-17), interleukin-6 (IL-6), and interleukin-22 (IL-22) promote immune-cell recruitment, stromal cell activation, and the clustering of B and T cells into organized aggregates (72, 73). In parallel, LTα/β-LTβR interactions between lymphoid tissue inducer-like cells (including B cells) and stromal cells help imprint a lymphoid organizer phenotype on local fibroblasts and endothelial cells. Chemokines including CXCL13, CXCL12, CCL21, and CCL19 then guide lymphocyte migration and compartmentalization, fostering the emergence of follicular-like zones within iBALT that resemble germinal centers (GCs) in SLOs and are predicted to support antigen-driven selection, affinity maturation, and memory B cell formation (70, 74, 75).

**Figure 1:**
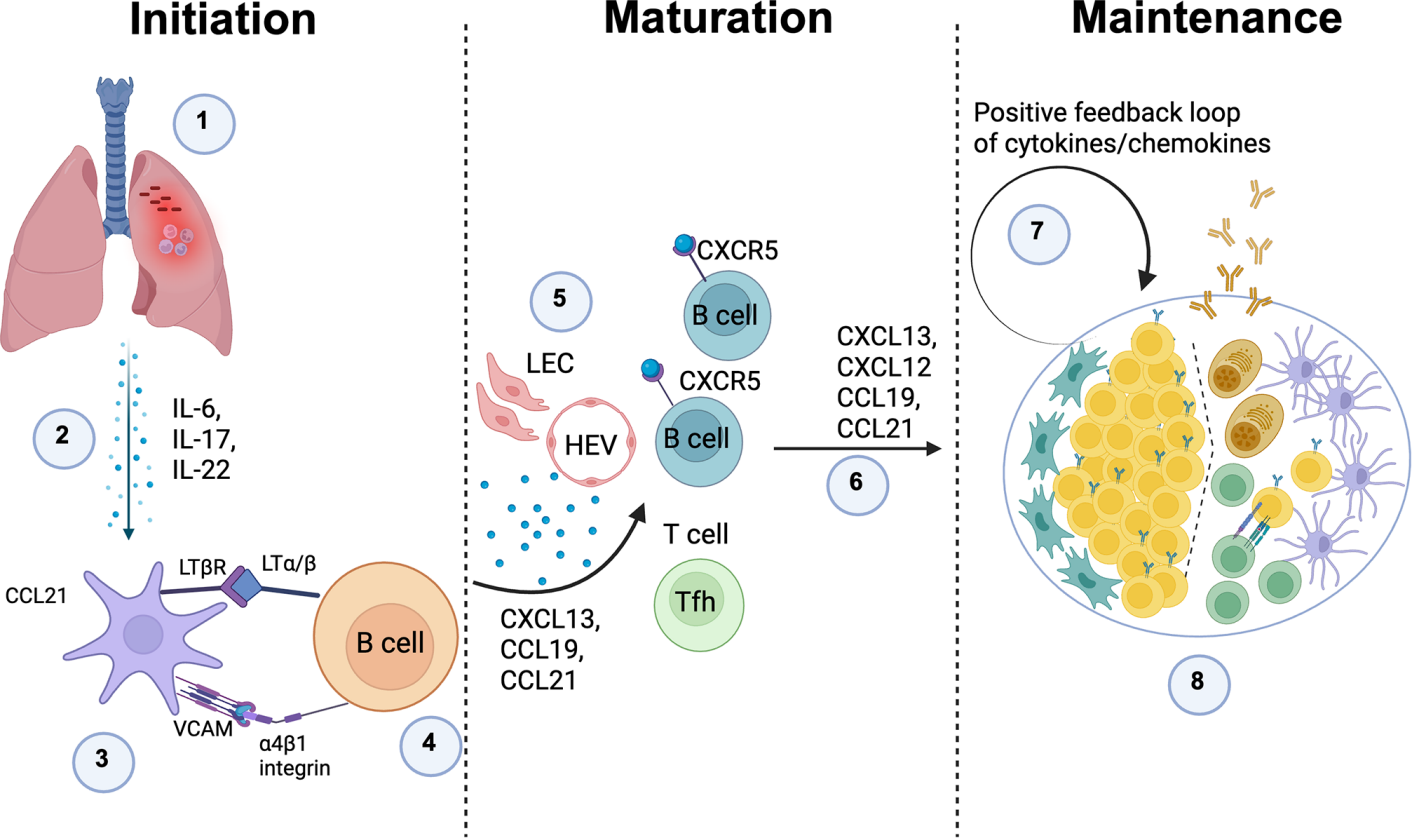
Putative mechanism of iBALT formation in TB: 1) *Mtb* infection leads to chronic inflammation; 2) Persistent inflammation drives the release of pro-inflammatory cytokines, including TNF family members, IL-6, IL-17, and IL-22, which promote immune cell recruitment and tissue remodeling; 3) Inflammatory cytokines activate lung stromal cells, prompting them to function as lymphoid tissue organizer (LTo) cells by upregulating LTβR and VCAM expression on their surface. This transformation is critical for TLS formation; 4) Activated B cells express LTα/β, which binds to LTβR on stromal cells, initiating their function as lymphoid tissue inducer (LTi) cells and promoting lymphoid-like structures; 5) LTα/β-LTβR interactions drive the expression of CXCL13, CCL19, and CCL21, which recruit B cells, T cells, and dendritic cells (DCs) to the inflamed site, creating a localized immune niche; 6) Production of CCL19, CCL21, CXCL12, and CXCL13, ensures the persistence of inducible bronchus-associated lymphoid tissue (iBALT) and reinforces local immune response; 7) A positive feedback loop amplifies the secretion of these critical chemokines and cytokines, stabilizing the iBALT structure and supporting its function as a persistent immune hub; 8) iBALT structures may develop germinal center-like formations, where T and B cells interact, leading to the generation of high-affinity antibodies against *Mtb* antigens, enhancing immune defense against infection. The image was created using Biorender. Define in the legend: Lymphatic endothelial cells (LEC), High endothelial venules (HEV).

Importantly, these LT cytokine-chemokine networks represent only one part of a broader, heterogeneous landscape of iBALT-inducing mechanisms. Studies in influenza, Pneumocystis infection, and LPS-driven neonatal inflammation show that iBALT can arise through both lymphotoxin-dependent and lymphotoxin-independent routes, with different cell types (e.g., Th17 cells, γδ T cells, ILC3, dendritic cells, and activated B cells) acting as lymphoid tissue inducer (LTi)-like populations, and with additional contributions from IL-1 family cytokines, type I interferons, and TNF-family members beyond LTα/β (76, 77). Moreover, iBALT may form *de novo* or via remodeling of pre-existing peribronchial/perivascular leukocyte-stromal aggregates, and the relative contribution of these pathways is likely shaped by pathogen type, antigen load, age, and the chronicity of inflammation (74, 76). Thus, the model below should be viewed as one experimentally supported framework rather than a single, exclusive mechanism of iBALT formation (**Figure 1**).

### 3.1 Initiation

A local inflammatory insult in the lung for example infection induces production of cytokines such as IL-6, IL-17 and IL-22. These signals activate perivascular/peribronchial stromal cells, which begin to express CCL21, VCAM-1 and LTβR. Circulating B cells expressing α4β1 integrin and membrane LTα/β are recruited to these sites and, through VCAM-integrin and LTα/β-LTβR interactions, start to imprint a lymphoid-organizer phenotype on the stromal cells, seeding early TLS aggregates.

### 3.2 Maturation

As the niche stabilizes, lymphatic endothelial cells (LEC) and high endothelial venules (HEV) are induced, enabling efficient entry and exit of lymphocytes. Activated stromal cells, follicular dendritic cell (FDC) precursors and fibroblasts secrete homeostatic chemokines CXCL13, CXCL12, CCL19 and CCL21, which attract CXCR5^+^ and CXCR4^+^ B cells and CCR7^+^ T cells, including T follicular helper cells (Tfh). This chemokine-guided influx and segregation of cells drives the emergence of distinct B cell follicles and T cell zones, giving the structure a secondary-lymphoid-organ like architecture.

### 3.3 Maintenance

In the mature TLS, ongoing antigen stimulation and cytokine/chemokine production establish a positive feedback loop. GC like B cell clusters, plasma cells, FDC networks and T cells are maintained within the structure, allowing sustained local antibody production and T cell help. Depending on the inflammatory context, similar stromal and cytokine circuits can also stabilize alternative TLS configurations, so this model represents one major pathway rather than the only mechanism by which TLS can form and persist.

## 4 Pathogenic versus protective iBALT

Depending on the context iBALT can be either protective or contribute to pathology. Perhaps the simplest example is in organ transplantation, where immune regulation by iBALT confers transplant success (78–80) but pro-inflammatory iBALT results in rejection (78). Similarly, iBALT is associated with protection in conditions where inflammation is required such as in response to infection (74), or it can exacerbate inflammation in response to self-antigens contributing to autoimmunity.

### 4.1 Protective iBALT

In most infectious contexts, iBALT is associated with protection (15, 20, 21, 25, 81–88). This is partly due to the production of high-affinity antibodies within iBALT, which neutralize pathogens and limit their spread. These responses are enhanced upon secondary exposure in the presence of iBALT (89, 90). In mice infected with influenza, iBALT was protective even in mice completely lacking secondary lymphoid organs (91). Conversely, NHP infants do not form iBALT, resulting in a lower antibody response and increased pulmonary damage (92). Additionally, iBALT has been associated with reduced inflammation in viral (82) and bacterial infections (83, 84).

**Figure 2:**
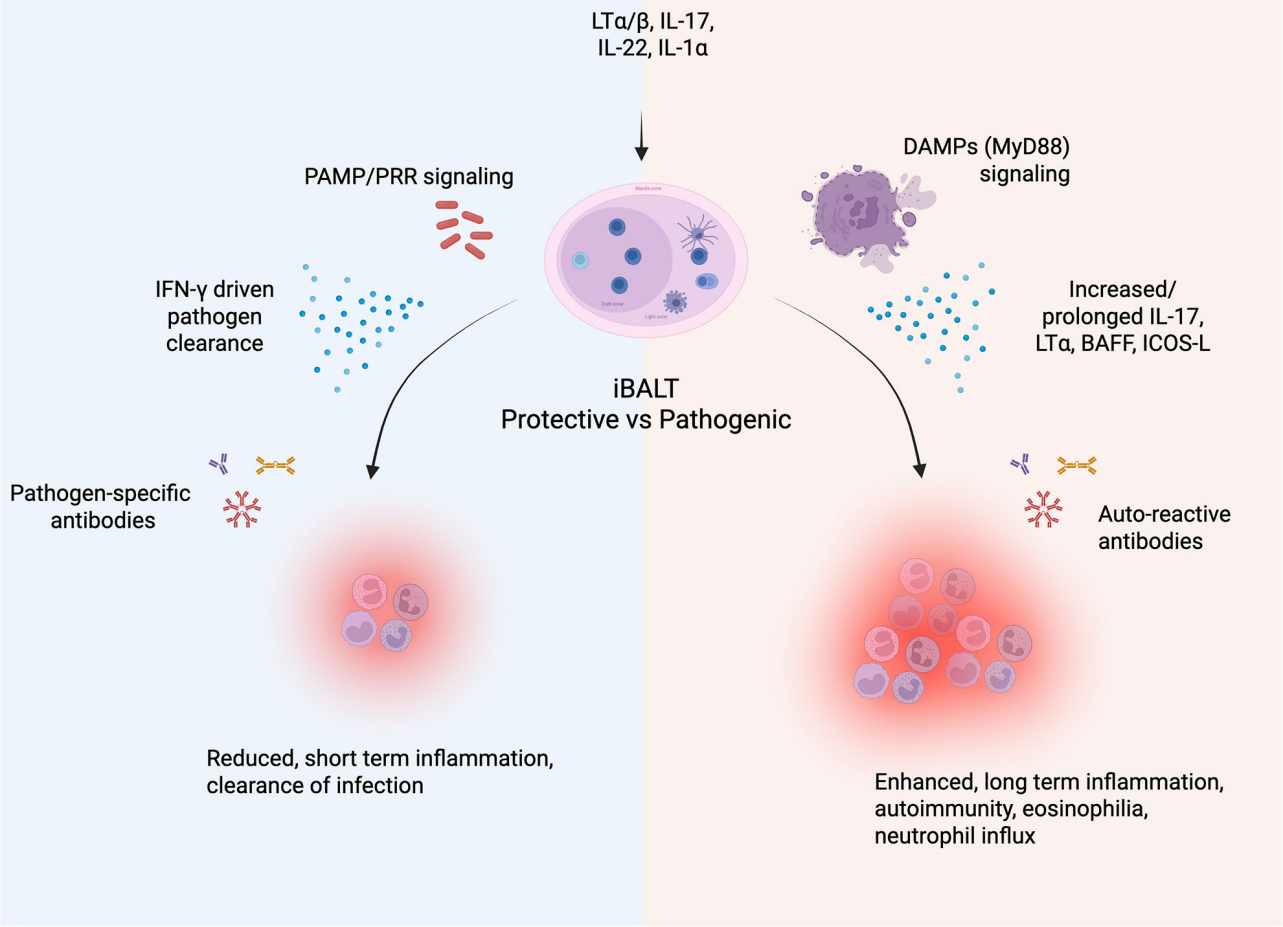
Description of protective vs pathogenic iBALT. Following the induction of iBALT, the local microenvironment can sway the response to be protective or pathogenic. In general, a protective response (blue) entails a specific response driven by PAMP/PRR signaling. This induces IFN-γ driven responses to clear the pathogen along with pathogen-specific antibodies, resulting in short-term inflammation. In contrast, a pathogenic response (red) is generally driven by DAMPs from local tissue damage accompanied by increased/prolonged IL-17, LTα, BAFF, ICOS-L, and auto-reactive antibodies. This leads to prolonged inflammation, eosinophilia, neutrophil influx, and autoimmune pathology. The image was created using Biorender.

Modified Vaccinia Ankara (MVA) and Pneumocystis infections highlight the significance of iBALT as a priming site against unrelated pathogens (85–87). In influenza models, local memory exhibits broader specificity compared to lymph node-selected memory (88). Additionally, iBALT has been linked to positive cancer outcomes (93–95). TLS have been shown to enhance CD4^+^ and CD8^+^ T cell responses in cancer patients (93), and the role of B cell-mediated priming of protective T cell responses is an active area of investigation in cancer immunotherapy. This parallels the protective responses observed in TB models, where iBALT formation is associated with antigen-specific CD4^+^ and CD8^+^ T cell responses (96) (**Figure 2****).**

### 4.2 Pathogenic iBALT

Induced hypersensitivity and persistent inflammation are often associated with detrimental iBALT formation. Continuous exposure to foreign substances, such as cigarette smoke, can induce lung inflammation, leading to chronic obstructive pulmonary disease (COPD). In this scenario, iBALT contributes to harmful inflammation associated with aging and inflammaging (97). Similarly, allergies involve persistent antigen stimulation, which can result in iBALT-associated immunopathology (98). Ongoing inflammation can also lead to a loss of tolerance and the development of autoimmune conditions such as Rheumatoid lung disease, Sjögren syndrome and pulmonary arterial hypertension (99). In these cases the sustained production of IL-17 and LT drives the formation of pathogenic iBALT (100). Consequently, elevated levels of BAFF, ICOS-L, and LTα drive B cell survival and auto-antibody production (75, 99, 101, 102), perpetuating the pro-inflammatory loop (**Figure 2**).

Respiratory syncytial virus (RSV) infection is a Th2 driven condition (103) where iBALT is linked to the disease (104). Interestingly, in Th2 and/or Th17 driven conditions (98, 105), allergies can further exacerbate RSV pathology (106). In graft versus host disease (GVHD), the recruitment of CXCR5^+^ Tfh and GC B cell worsens the disease (78), whereas recruitment of CXCR5^+^ Tregs reduces lung rejection (79). Notably, the recruitment of FoxP3^+^ Tregs into iBALT can prevent transplant rejection by inhibiting CD40 and CD28 signaling, and even permits multiple transplantations (80).

### 4.3 B cell heterogeneity as a driver of protective and pathogenic outcomes

Across infections, whether B cells are protective or pathogenic depends on which B cell subsets are engaged and the affinity/quality of the antibodies they produce. In well-organized GCs in lymph nodes, spleen, or iBALT, follicular and GC B cells undergo somatic hypermutation and stringent selection; high-affinity clones are positively selected into the memory and plasma-cell compartments, where they produce class-switched antibodies that efficiently neutralize pathogens and provide durable protection after infection or vaccination (107, 108). By contrast, extrafollicular plasmablasts and “atypical” or age-associated B cells often expand under conditions of high or persistent antigen load and strong inflammatory signals. In severe COVID-19, for example, dominant extrafollicular B cell responses are associated with robust but relatively low-mutation antibody production, lupus-like autoreactivity, and poor clinical outcomes, indicating how impaired affinity maturation can track with immunopathology (109). Bregs add another layer of complexity as IL-10/IL-35 producing Bregs can limit tissue damage in autoimmunity and some infections, but in chronic viral disease they may also suppress protective T cell responses and favor pathogen persistence (110, 111). In addition, as mentioned, marginal zone B cell, which are present in TB infected lung tissue (14), secrete many relevant cytokines following *Mtb* stimulation. Antibody quality then modulates these subset effects: high-affinity, strongly neutralizing antibodies from well-selected B cells are typically protective, whereas non- or sub-neutralizing antibodies generated by sub optimally selected B cells can form immune complexes or mediate antibody-dependent enhancement (ADE), as shown for dengue and other flaviviruses where intermediate titers of cross-reactive IgG can increase viral entry and drive severe disease via Fcγ-receptor–bearing myeloid cells (112).

Although detailed mechanistic data in TB are more limited, human and experimental studies already suggest a similar spectrum. B cell rich TLS/iBALT adjacent to granulomas contain GC-like B cells and Tfh cells and are often associated with better-organized lesions and evidence of local immune control, consistent with a protective role for well-selected, tissue-resident B cell responses and their antibodies (20, 113–115). Conversely, B cells can also promote granulomatous inflammation and lung damage in chronic murine TB, highlighting that dysregulated or chronically stimulated B cell compartments may tilt toward pathology (16). This protective versus pathologic balance likely depends not only on the presence of B cells, but also on how their activation is focused and selected within these local niches. This raises the possibility that *Mtb* actively perturbs B cell selection upstream of antigen specificity to limit adaptive immunity and/or drive inflammation and tissue destruction to enhance transmission. Several studies have shown that active TB leads to the expansion of antibodies targeting unrelated pathogens, including measles, RSV and tetanus (116, 117). The mechanism is unclear, but one intriguing possibility is the existence of superantigen-like immune modulation (118) by a TB derived product. This has been described for other bacterial pathogens and involves bacterial factors binding conserved immunoglobulin regions outside the complementarity determining regions (CDRs) to broadly engage BCRs and drive non-cognate activation, deletion, and repertoire distortion (119). Such broad BCR pressure could reshape the pool of B cells available for iBALT GC reactions and reduce the probability of generating high-affinity protective clones where they are needed most. However, while T cell-directed superantigen activity has been proposed in TB (118), no *Mtb*-derived B cell superantigen has been identified to date, and similar patterns of non-cognate B cell activation could also arise from alternative mechanisms such as innate receptor signaling and inflammatory cytokine milieus (120).

Taken together, these examples support a model in which (i) subset composition (GC vs extrafollicular vs regulatory vs tissue-resident B cells) and (ii) antibody affinity/function (neutralizing vs sub-neutralizing, pro-inflammatory Fc profiles) jointly determine whether B cell responses are net protective or pathogenic.

## 5 iBALT in TB

Accumulating evidence suggests a positive correlation between iBALT formation and improved control of *Mtb* and latent TB infection (LTBI) (81, 121, 122). The structural organization of iBALT facilitates close interactions between B cells, T cells, and APCs thereby promoting efficient antigen presentation and T cell priming. Specifically, B cells within iBALT can present *Mtb* antigens to CD4^+^ T cells, supporting their activation and differentiation into effector T cells that secrete IFN-γ and other cytokines essential for macrophage activation and bacterial clearance (40). This interaction between B cells and T cells within iBALT may significantly enhance immune efficacy, particularly in the early stages of *Mtb* infection when localized immune activation is critical for controlling bacterial replication (15, 40).

The formation of lymphoid follicles is observed not only in *Mtb* infection but also following mucosal vaccination, which confers superior protection against TB in animal models (22, 123–125). Non-human primate studies have shown that macaques vaccinated with *Mtb*ΔsigH demonstrate protective immunity, characterized by the absence of granulomas and the formation of iBALT, along with strong antigen-specific CD4^+^ and CD8^+^ T cell responses (22). Intriguingly, exposure to commensal gut bacteria confers protection to pneumonia in mice by priming ILC3 cells to migrate to the lung (126). ILC3s mediate an early protective role against *Mtb* infection by priming iBALT formation via IL-22 and IL-17 and thereby starting the CXCL13/CXCR5 cascade (127). Finally, oral exposure to non-tuberculous mycobacteria promotes iBALT formation in BCG vaccinated mice and subsequent protection (15). Similarly, delivery of *Mtb* antigen-primed dendritic cells (DCs) rapidly induced iBALT formation and near-sterilizing immunity in mice (128). Collectively, these findings highlight that the induction of local lymphoid structures and B cell responses at mucosal sites is a critical component of protective immunity against TB and represents a promising strategy for next-generation vaccines.

In humans, iBALT is linked to latency and containment of infection, whereas its absence is associated with active disease (81, 121). In NHP, the presence of iBALT is associated with protection (22, 129). Recently intravenous-BCG administration achieved complete protection in NHP, characterized by the distinct appearance of CD11c^+^ and CD3^+^ immune infiltrates, termed “microgranulomas”, which are associated with protection (96). A Th1/Th17 response also correlated with protection (130). An elevated TB-specific IgM response was observed in infected human lungs (14), and a robust IgM response was linked to protection in NHP (131). While B cell depletion had a modest effect on protection in this model, protection primarily relies on CD4 and CD8 immunity (132), again suggesting that iBALT may be an important mediator of protection.

While iBALT is generally associated with protective immunity in TB, it may also contribute to disease progression in certain cases (as described above), particularly when linked to chronic inflammation. A study by Chen et al. found B cells to enhance inflammation and host-detrimental immunopathology (16) by promoting Th1 responses while restricting anti-inflammatory IL-10. In the absence of B cells, mice survived *Mtb* infection significantly longer. Differences in study outcomes may be due, in part, to the use of different *Mtb* strains, as variations in pathogen characteristics—such as cell wall components and lipid moieties—can shape the host immune response and influence the balance between protective and harmful inflammation. Moreover, the formation and function of iBALT may be influenced by host factors such as genetic susceptibility, sex, age, or co-morbidities, all of which can impact the efficacy of the immune response. In particular, sex has been shown to have a profound impact on the immune system and may therefore significantly shape iBALT formation.

## 6 Sex differences in immunity and iBALT formation

Sex differences in the immune system arise from variations in the development, function, and regulation of diverse immune cell populations, shaping distinct responses in males and females. These differences are influenced by a combination of genetic, hormonal, and environmental factors and have implications for immunity, autoimmunity, and vaccine responses (133). The underlying reasons for sex differences in TB are not fully understood and were long thought to result from gender-related differences in exposure, behavior, and lifestyle (26, 27). The study of the role of biological sex in TB is relatively recent and is based, in part, on the observation that the male bias in TB incidence becomes apparent only after puberty (WHO report) —indirectly implicating sex hormones as potential key mediators of this sex difference. Indeed, growing experimental evidence supports a direct impact of biological sex on anti-TB immune responses. In particular, murine infection models, which allow the isolation of biological sex from gender-related confounders, consistently demonstrate increased susceptibility of males compared with females. Male mice exhibit significantly reduced survival following *Mtb* infection, and this phenotype is observed across different genetic backgrounds (e.g. C57BL/6 and BALB/c mice) and with two distinct *Mtb* strains (25, 134, 135). Non-castrated male mice exhibited significantly higher mortality rates and bacillary burdens compared with female and castrated male mice, supporting a role for testosterone in increased disease susceptibility (134). In addition, BCG-vaccinated female mice showed greater protection against TB than males in two mouse models (136, 137). Collectively, these results indicate that females develop superior protective immunity against *Mtb* compared with males. However, this is not to discount the contribution of non-biological factors to the increased risk of TB disease in human males, which is likely to be significant.

Males and females exhibit important sex-specific differences in the B cell compartment, including variations in B cell numbers, activation status, and antibody production. Females generally display higher B cell counts and mount stronger humoral immune responses, producing higher antibody levels following infection or vaccination (133). Consistent with this, recent studies suggest notable sex differences in the formation, structure, and function of iBALT. In COPD, the development of lymphoid follicles is more pronounced in female mice upon exposure to cigarette smoke (138). These differences were abolished with ovariectomy, suggesting that female sex hormones play a key role in lymphoid follicle development. While their exact role in COPD remains unclear, lymphoid follicles have been linked to severe disease progression (139). Notably, females appear to be more susceptible to developing COPD and exhibit increased expression of lymphoid follicles in the airways of COPD-affected lungs (138, 140). Similarly, in a model of collagen-induced arthritis (CIA), Dimitrijević et al. (2020) (141) observed sex differences in the generation of T follicular helper cells (Tfh), a subset of CD4^+^ T cells crucial for B cell responses and germinal center formation. Female rats, who are more susceptible to CIA, exhibited significantly higher frequencies of Tfh cells, resulting in stronger GC B cell formation, elevated antibody responses, and more severe disease progression compared to males (141). In contrast, *Mtb* infected female mice –who exhibit improved long-term control of *Mtb* infection compared to males– develop larger B cell follicles than their male counterparts (25). The smaller size of B cell follicles in males, which is associated with increased disease progression and higher mortality rates, underscores the protective role of B cell follicles in TB immunity. Several cytokines critical for the formation and maintenance of iBALT are upregulated in females compared to males across various diseases (**Table 1**). Importantly, cytokines essential for protective immunity in TB, such as IL-1α, IL-6, IL-23, and IL-17 (156–159) play a crucial role in the induction and formation of iBALT and are elevated in *Mtb*-infected females compared to males. More recently, Swanson et al. (2023) demonstrated a crucial role of antigen-specific B cells in the strategic localization of Tfh-like cells within iBALT to mediate *Mtb* control (40). According to Linge et al., iBALT structures in TB-susceptible mice were populated by B cells lacking TB antigen specificity, underscoring once again the critical role of antigen-specificity in mounting a protective iBALT-mediated immune response (160). However, the influence of the biological sex on the generation of Tfh cells and the close interaction with B cells in iBALT in TB has yet to be elucidated.

**Table 1:** Sex differences in iBALT formation.

iBALT formation	disease	species	Sex	References
Cytokines				
CXCL13/ CXCR5	TB	Mouse	No differences	(26)
CCL19/ CCL21/CCR7	TB	Mouse	Increased in females	(26)
IL-7	CIA	Mouse	No differences	(141)
IL-17	CIA TB UTI Influenza	Mouse Mouse Mouse Mouse	Increased in females	(26, 141–143)
IL-1α	TB	Mouse	Increased in females	26
IL-6	TB SLE Influenza Influenza (vaccination)	Mouse Mouse Mouse Human	Increased in females	(143–146)
IL-21	CIA SLE	Mouse Mouse	Increased in females	(141, 147)
Type I IFN	SLE HIV COVID-19 Hanta (Seoul)	Human Human Human Rat	Increased in females	(148–151)
IL-23	TB	Mouse	Increased in females	(26)
IL-27	CIA	Mouse	Increased in females	(141)
IL-2	CIA	Mouse	Increased in males	(141)
BAFF	COPD SLE	Mouse/Human Mouse/Human	Increased in females	(138, 152)
Cell types				
B cells	–	Human	Increased in females	(133, 153, 154)
CD4^+^ T cells	–	Mouse/Human	Increased in females	(133, 155)
Tfh cells	CIA SLE	Mouse Mouse	Increased in females	(141, 144, 147)

TB, tuberculosis; CIA, collagen-induced arthritis; UTI, urinary tract infection; SLE, systemic lupus erythematosus; COPD, chronic obstructive pulmonary disease.

**Figure 3:**
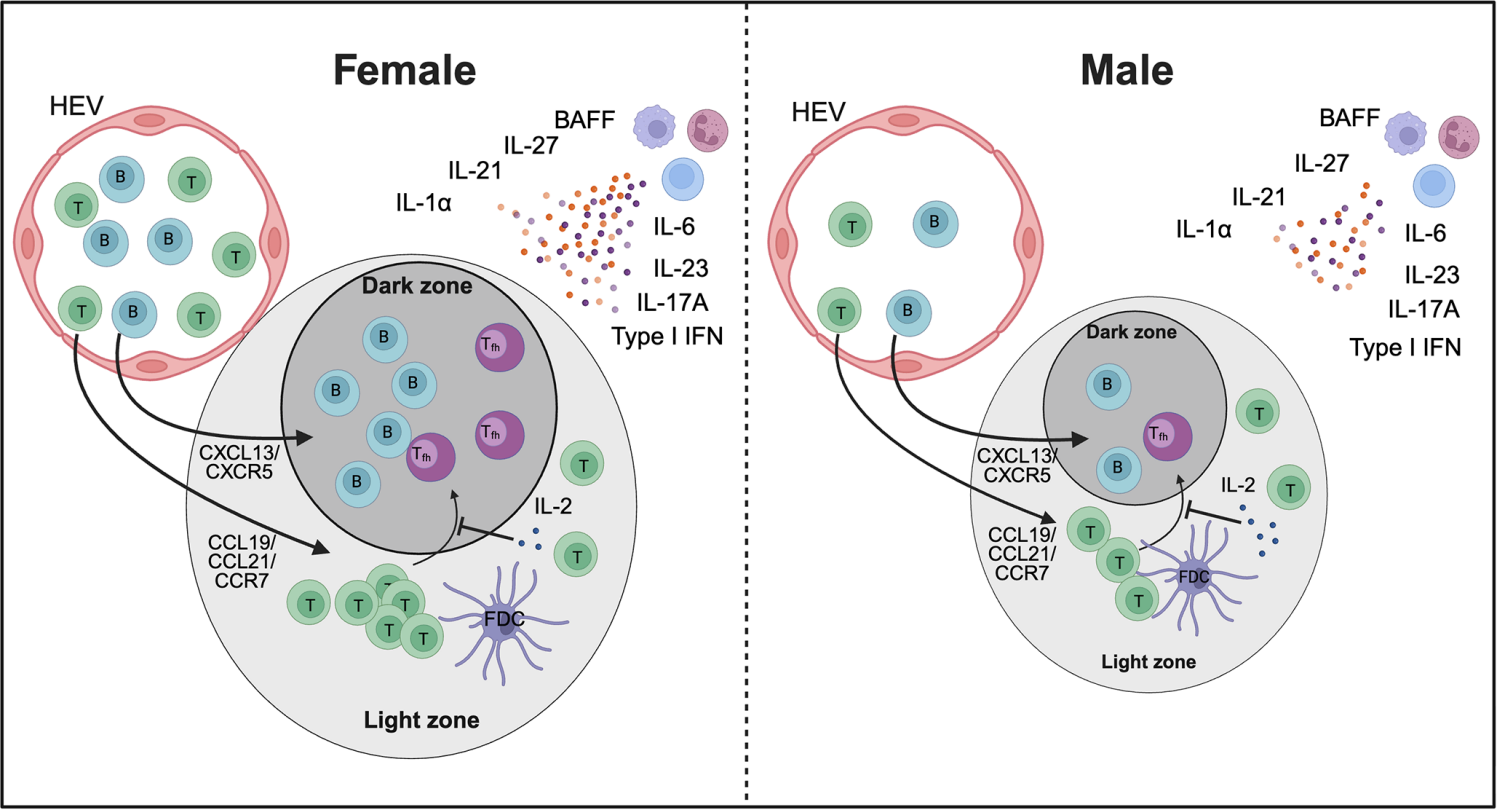
Proposed model of sex differences in iBALT formation. Higher numbers of B cells and T cells, particularly CD4^+^ T cells, along with increased levels of cytokines involved in iBALT development, may promote greater recruitment of these cells in females compared to males, potentially resulting in larger iBALT structures. In addition, elevated IL-21 and IL-27 together with reduced IL-2 could enhance Tfh cell differentiation in females. Together, these mechanisms may contribute to sex-specific differences in immunity, autoimmunity, and vaccine responses. HEV - high endothelial venules. The image was created using Biorender.

In summary, sex-specific differences in iBALT formation (**Figure 3**) contribute to the generally more robust immune responses in females, which may play an important role in the defense against infections like TB, but also in the higher prevalence of certain autoimmune and inflammatory diseases in the female population.

## 7 Discussion and concluding remarks

B cells play a far more dynamic and essential role in TB immunity than previously appreciated. Their involvement in antigen presentation, cytokine secretion, and TLS formation suggests that they are integral to shaping local immune responses in the lung. The discovery of iBALT as a potential site of protective immune activation challenges the conventional T cell-centric view of TB immunity and highlights new opportunities for immunotherapeutic strategies. The dual nature of iBALT—as both a protective structure and a contributor to pathology—necessitates a nuanced understanding of its regulation. Notably, IL-10-producing regulatory B cells may play a pivotal role in modulating TB immunity. While some B cell subsets contribute to protective responses, IL-10-secreting B cells could suppress pro-inflammatory immune activation, potentially limiting excessive lung damage but also creating an immunoregulatory environment that favors persistent *Mtb* infection. The balance between protective and immunosuppressive B cell functions within iBALT may be critical in determining TB disease outcomes.

Building on the evidence summarized in this review, we propose that B cells and iBALT could advance TB vaccine development and immunotherapy through several mechanisms. iBALT forms a lung-resident, germinal center-like niche where B cells undergo antigen-driven selection, class switching, and affinity maturation at the site of *Mtb* infection, producing high-affinity IgA and IgG that opsonize bacilli, neutralize secreted factors, and engage Fc-mediated effector pathways. B cells also act as antigen-presenting cells and cytokine producers, shaping local Th1, Th17, and Tfh responses and granuloma organization. Next-generation TB vaccines could be designed to induce protective iBALT using mucosal delivery platforms, adjuvants that promote lymphoid neogenesis and CXCL13-dependent recruitment, and antigens efficiently captured and presented by B cells, thereby establishing long-lived, tissue-resident B and T cell immunity in the lung. In parallel, defining B cell antigenic specificities, isotypes, and functional profiles in controlled versus progressive disease can guide selection of *Mtb* antigens and antibody subclasses for monoclonal therapies to enhance clearance, shorten chemotherapy, or prevent reactivation. This strategy may also identify targets for host-directed interventions that modulate B cell or iBALT function to reinforce protective rather than pathological lung immunity.
